# Patterns of Dorsal Fin Shape Variation in Commerson’s Dolphins Across Isolated Subantarctic Populations

**DOI:** 10.3390/ani16162510

**Published:** 2026-08-12

**Authors:** Franco Cruz-Jofré, María José Pérez-Álvarez, Analía San Martín, Frederick Toro, Juan Capella, Jorge Gibbons, Carlos Olavarría, Jorge Acevedo, Elie Poulin, Laura M. Pérez, Manuel J. Suazo, Alexis Matheu, Hugo A. Benítez

**Affiliations:** 1 Facultad de Recursos Naturales y Medicina Veterinaria, Universidad Santo Tomás, Viña del Mar 2561780, Chile; francocruzjo@santotomas.cl (F.C.-J.); fredericktoroc@gmail.com (F.T.); 2 Millenium Institute Biodiversity of Antarctic and Subantarctic Ecosystems (BASE), Santiago 7800003, Chile; epoulin@uchile.cl; 3 Departamento de Ciencias Ecológicas, Facultad de Ciencias, Universidad de Chile, Santiago 8330111, Chile; 4 Centro de Investigación Eutropia, Santiago 8320000, Chile; 5 Museo Acatushún de Aves y Mamíferos Marinos (AMMA), Proyecto IMMA, Ushuaia 9410, Argentina; analiasmn@gmail.com; 6 Panthalassa Non-Governmental Organization, Red de Estudios de Vertebrados Marinos en Chile, Santiago 8320000, Chile; 7 Fundación Whalesound, Punta Arenas 6200000, Chile; jjcapella@yahoo.com; 8 Instituto de la Patagonia, Universidad de Magallanes, Punta Arenas 6210427, Chile; jorge.gibbons@umag.cl; 9 Centro de Estudios Avanzados en Zonas Áridas (CEAZA), La Serena 1700000, Chile; carlos.olavarria@ceaza.cl; 10 Centro de Estudios del Cuaternario Fuego-Patagonia y Antártica (Fundación CEQUA), Punta Arenas 6200000, Chile; jorge.acevedo@cequa.cl; 11 Departamento de Ingeniería Industrial y de Sistemas, Universidad de Tarapacá, Arica 1000000, Chile; lperez@academicos.uta.cl; 12 Vicerrectoría de Investigación y Postgrado, Universidad de La Serena, La Serena 1700000, Chile; suazo.mj@gmail.com; 13 Centro de Investigación Institucional, Universidad Bernardo O’Higgins, Santiago 8370993, Chile; alexis.matheu@ubo.cl; 14 Instituto One Health, Facultad de Ciencias de la Vida, Universidad Andrés Bello, República 440, Santiago 8370035, Chile; 15 Cape Horn International Center (CHIC), Centro Universitario Cabo de Hornos, Puerto Williams 6350000, Chile; 16 Centro de Investigación de Estudios Avanzados del Maule, Universidad Católica del Maule, Talca 3466706, Chile

**Keywords:** geometric morphometrics, dorsal fin morphology, phenotypic variation, cetaceans, population differentiation

## Abstract

Understanding morphological variation across geographic space is central to evolutionary and functional studies of marine vertebrates. Here, we examine variation in dorsal fin shape in the Commerson’s dolphin (*Cephalorhynchus commersonii*), a small coastal cetacean with a disjunct distribution across South America and the Kerguelen Islands. Dorsal fin shape was quantified using geometric morphometrics based on photographs, and comparisons were conducted across subspecies and populations. Principal Component Analysis revealed partial overlap in morphospace, with higher shape variability in the Kerguelen subspecies. Canonical Variate Analysis showed separation among geographic groups, although patterns of overlap remained. Differences in mean fin shape were observed among populations, particularly in the relative development of the free span and base of the fin. Kerguelen dolphins exhibited a relatively broader fin base and reduced free span, whereas South American populations generally showed a wider free span, with additional variation among regional populations. Morphological patterns did not fully correspond to population genetic structure, suggesting that multiple factors may contribute to phenotypic variation in this species. Similarities between geographically distant populations highlight the potential influence of shared environmental conditions. These results emphasize the value of dorsal fin morphology for investigating spatial variation in small cetaceans and provide a basis for future integrative studies combining ecological, physiological, and genetic data.

## Simple Summary

Commerson’s dolphin occurs in geographically isolated populations across South America and the Kerguelen Islands in the southern Indian Ocean. We examined whether dorsal fin shape varies among these populations using geometric morphometric methods. Our results revealed differences in fin shape between subspecies and populations, although these patterns did not fully match previously described genetic structure. Interestingly, dolphins from distant regions with similar environmental conditions showed comparable fin shapes. These findings suggest that multiple factors, including environmental conditions, may contribute to morphological variation in this species and highlight the usefulness of dorsal fin morphology for studying population differentiation in cetaceans.

## 1. Introduction

Understanding patterns of morphological variation across geographic space is central to evolutionary biology and functional morphology. In marine vertebrates, spatial heterogeneity in environmental conditions, population structure, and ecological interactions can contribute to phenotypic differentiation among populations [1,2]. In cetaceans, morphological traits such as dorsal fins are particularly relevant, as they are associated with locomotion and hydrodynamic stability [3]. Evaluating variation in these structures provides an opportunity to explore how morphology differs across populations without assuming a priori adaptive frameworks. Marine environments are characterized by strong spatial and temporal variability in oceanographic conditions, including temperature, productivity, and circulation patterns [4,5]. These factors can influence the distribution, ecology, and phenotypic variation in marine organisms, particularly in species with broad geographic ranges or discontinuous distributions [6,7,8]. In cetaceans, oceanographic processes such as currents and regional productivity regimes can shape habitat use and population structure [1,2]. Appendicular structures play key roles in multiple aspects of cetacean biology. In dolphins, dorsal and pectoral fins primarily function as stabilizers during swimming, contributing to hydrodynamic efficiency [3]. Beyond locomotion, dorsal fins are also involved in physiological processes, particularly thermoregulation, due to their vascularization and potential role in heat exchange. Dorsal fins are also involved in physiological processes, including thermoregulation, due to their vascularization and potential role in heat exchange. In odontocetes, vascular networks within the fin have been described as part of counter-current systems that may contribute to thermal regulation [9,10]. These multiple functional roles highlight the potential relevance of dorsal fin morphology as a trait subject to variation across populations. Morphological variation in cetaceans has been widely documented across both external and skeletal traits, often revealing patterns of differentiation among populations and ecological contexts. For instance, studies on bottlenose dolphins have demonstrated consistent variation in skull morphology at broad geographic scales, with coastal populations often exhibiting greater morphological variability compared to more uniform offshore counterparts [8]. Morphological variation in cetaceans has been documented in both cranial and external traits. Skull morphology has revealed geographic and ecological differentiation among populations and ecotypes [7,8], whereas dorsal fin morphology has also been shown to vary among populations, reflecting a combination of hydrodynamic, behavioural, and environmental influences [11]. Together, these studies suggest that external morphology can provide valuable information on population differentiation in cetaceans. Together, these findings highlight that morphological traits in cetaceans may vary substantially within species, providing a useful framework for examining phenotypic differentiation across geographically structured populations. Within the genus *Cephalorhynchus*, species diversification has been associated with Pleistocene glacial cycles in circumpolar waters [12], resulting in geographically isolated distributions across the Southern Hemisphere. This pattern suggests a gradual eastward colonization from Oceania toward South America and the Indian Ocean (the latter, consistent with the Long Dispersal and Establishment Model, LDDE; Crisp et al. [13]). *C. commersonii* is currently distributed in coastal and subantarctic waters from central Argentina to southern South America (including the Strait of Magellan, Tierra del Fuego, and the Malvinas/Falklands Islands), with isolated populations in the Kerguelen Islands, Indian Ocean [14]. This distribution spans a wide range of oceanographic conditions, particularly in sea surface temperature (SST). Across its range, SST varies markedly. In the Atlantic north of 25° S, SST averages above 21 °C, where the Brazil Current flows southward and collides with the Malvinas/Falklands Current near 38° S before veering offshore. On the continental shelf north of Puerto Madryn and Puerto Rawson, coastal waters typically range from 10 to 15 °C [15], with autumn–winter temperatures fluctuating between 5.8 and 8.2 °C [16]. Along the coast between 42° S and 47° S, SST ranges from 7.7 to 15.5 °C. In the southernmost range of *C. commersonii*, the Antarctic Circumpolar Current and the Malvinas/Falklands Current run along the outer continental slope. In coastal waters near the Malvinas/Falklands Islands, summer SST varies between 7 and 12 °C [17], whereas in the Strait of Magellan summer temperatures range from 3.7 to 4.0 °C [18], although the most commonly observed temperatures are approximately 5.8–9.9 °C [19,20]. However, the lower range values are comparable to those reported for the Kerguelen Islands (3.39–4.96 °C during October–April, 1982–2015; [21]. South American and Kerguelen populations occupy contrasting oceanographic settings. On the Southwestern Atlantic shelf, sea surface temperature is strongly seasonal and influenced by the Brazil– Malvinas/Falklands confluence and shelf upwelling, with typical values of 7.7–15.5 °C between 42° S and 47° S and higher averages north of 25° S [15,16,22]. In contrast, Kerguelen populations inhabit cold circumpolar waters under the direct influence of the Polar Front, where summer SST generally ranges from about 3 to 5 °C and productivity is elevated due to persistent mixing and upwelling. These differences in thermal regime, current systems, and primary productivity characterize distinct oceanographic contexts across the species’ range. Two subspecies have been described [23]: the Kerguelen subspecies (*C. commersonii kerguelenensis*), which inhabits circumpolar waters, and the South American subspecies (*C. c. commersonii*). These contrasting distributions, together with distinct oceanographic conditions, make this species a suitable system for exploring morphological variation across geographically isolated populations. In this context, we examined dorsal fin shape variation in *Cephalorhynchus commersonii* across its subantarctic distribution using geometric morphometrics. Specifically, we compared dorsal fin morphology between the two recognized subspecies (*C. c. commersonii* and *C. c. kerguelenensis*) and among the main geographic population units previously identified from genetic studies. We expected geographically isolated populations to exhibit significant differences in dorsal fin shape, reflecting their evolutionary divergence. However, because some populations occupy oceanographic environments with similar thermal conditions despite their genetic isolation, we also expected that morphological similarity would not necessarily parallel population genetic structure, suggesting that environmental conditions may contribute to shaping dorsal fin morphology.

## 2. Materials and Methods

### 2.1. Study Area

The study encompassed the species’ full range and focused on four geographic sectors: northern Argentine Patagonia (40–43° S), the Strait of Magellan in Chile (52–54° S), and the Malvinas/Falklands Islands (50–52° S) in the South Atlantic Ocean; and the Kerguelen Islands in the French Southern and Antarctic Lands, southern Indian Ocean (49° S).

### 2.2. Data Acquisition

Dorsal fin: Dorsal fin photographs of both subspecies of *Cephalorhynchus commersonii* in strict lateral view were obtained from three sources (Table 1):

1. Images taken from boats during cetacean surveys conducted by the authors themselves.
2. Photographs available on iNaturalist under the Attribution-NonCommercial license and flagged as Research Grade (https://www.inaturalist.org/taxa/41513-Cephalorhynchus/browse_photos?photo_license=cc-by-nc; accessed 15 January 2026).
3. Images from the Photo-identification Catalogue of Commerson’s Dolphins of the Kerguelen Islands French Southern and Antarctic Territories produced by the Centre d’Études Biologiques de Chizé, CNRS [24].

Digital photographs from iNaturalist were used to fill gaps in under-sampled areas. The platform’s citizen science framework and curation standards enable reliable data use [25]. The species is conspicuous, with an unmistakable coloration pattern relative to those of other small cetaceans in the region, which facilitates accurate identification from online photographs. To ensure comparability among image sources and minimize digitizing errors, only photographs showing the dorsal fin in strict lateral view and approximately perpendicular to the body axis were included. Images were further screened for adequate focus, clarity, contrast, viewing angle, and minimal perspective distortion prior to landmark digitization [26]. Photographs of both subspecies were considered, distinguished by their geographic origin and dorsal coloration. All photographs included locality information.

**Table 1:** Sampling groups in dorsal fin analysis, localities, sample size (N), and data sources. Group definitions follow Kraft et al. (2021) [27] for populations. NP = North population (Argentina), SM = Strait of Magellan (Chile), and Robineau et al. (2007) [23] for subspecies.

Group	Subspecies	Locality	N Dorsal Fin (Source)
Argentina NP	*C. c. commersonii*	North population: Punta Delgada, Puerto Rawson, Bahía Camarones, Río Deseado, Puerto San Julián	9 (iNaturalist)
Malvinas/Falklands Is.		Coastal water in Malvinas/Falklands islands Southern Atlantic	4 (iNaturalist)
Chile SM		Strait of Magellan, Chile, subantarctic region	33 (iNaturalist, and authors of photographs)
Kerguelen Is.	*C. c. kerguelensis*	Island Kerguelen, subantarctic region	38 (Tixier et al., 2015) [24]

### 2.3. Data Analysis of Dorsal Fin Shape

**Figure 1:**
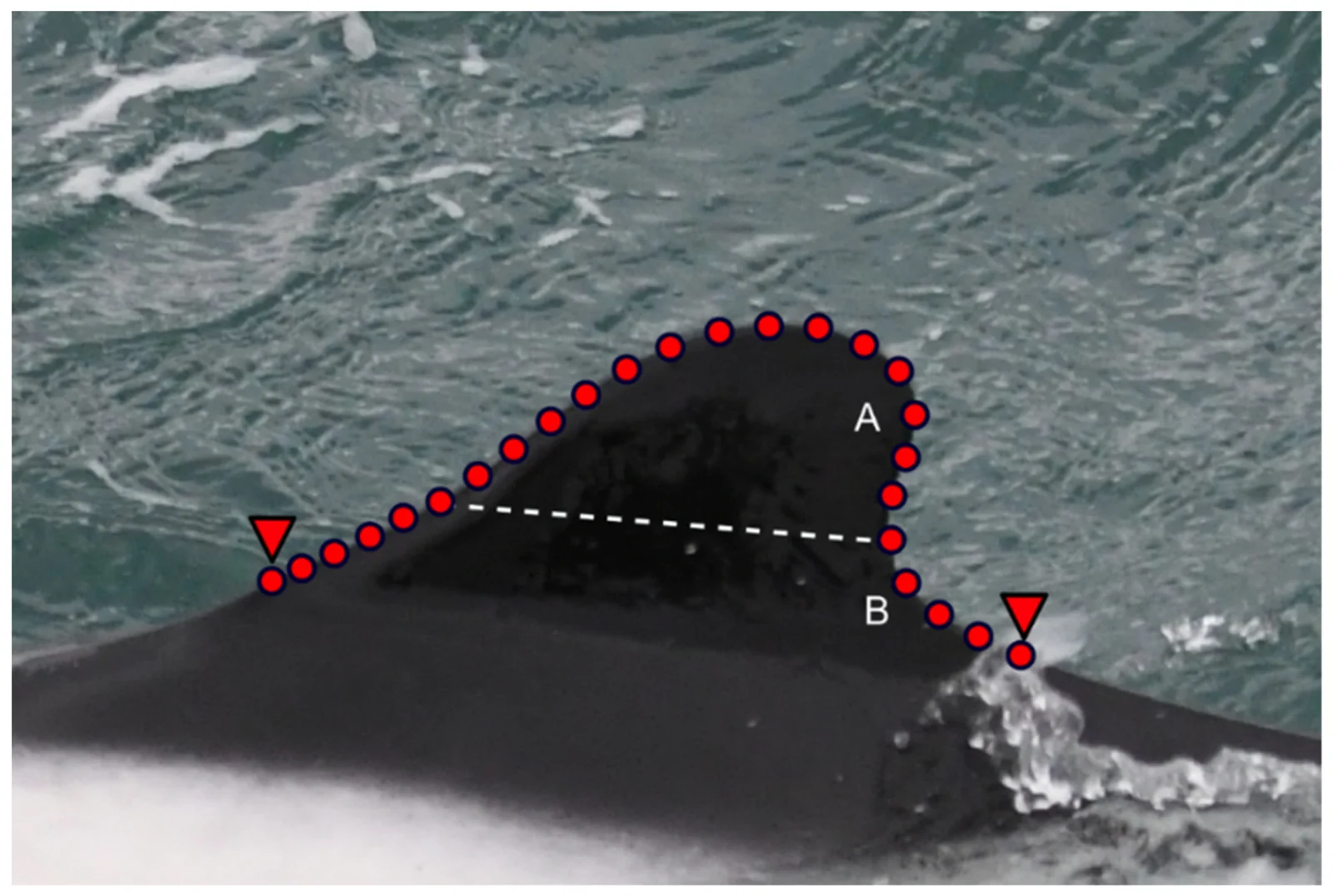
Configuration of 25 landmarks and semilandmarks used to characterize dorsal fin shape in *C. commersonii*. Red triangles indicate the anterior and posterior insertion points of the dorsal fin. The letters A and B identify the two anatomical regions of the dorsal fin separated by the dashed line: A, free span; B, fin base.

Geometric morphometric procedures based on landmarks were applied to analyze dorsal fin shape, which was examined in left lateral view. Twenty-five landmarks and semilandmarks were identified to characterize dorsal fin shape, including the anterior and posterior insertion points of each fin (see Figure 1). Semilandmarks were placed at equidistant intervals with TPSdig2 [28]. The number of landmarks was set to provide complete coverage of the fin margin. The start and end points corresponded to clearly delimited folds at the fin base. In addition, 11 basal landmarks were defined to distinguish fin regions, namely the free span (A) and the fin base (B).

Individuals were classified into two subspecies following the geographic distributions described by Robineau, Goodall, Pichler and Baker [23]: *Cephalorhynchus commersonii commersonii* from South American coasts (*n* = 46) and *C. c. kerguelenensis* from the Kerguelen Islands *(n* = 38). In addition, Commerson’s dolphins were compared according to the population genetic structure proposed by Kraft et al. [27], which recognizes three geographic units in South America and one in the Kerguelen Islands that differ in their degree of connectivity (see Table 1).

A generalized Procrustes superimposition was performed to align landmark configurations and remove non-shape variation [29]. From the aligned coordinates, a covariance matrix of shape variables was computed and subjected to Principal Component Analysis (PCA) to visualize the major axes of dorsal fin morphological variation among groups [30]. This approach allowed us to summarize multivariate shape variation into orthogonal axes, facilitating the identification of group-specific patterns and overlap in morphospace. To enhance group separation and better visualize shape differences, a Canonical Variate Analysis (CVA) was conducted [31].

Shape differences among groups were assessed using Procrustes coordinates derived from the generalized Procrustes superimposition. Patterns of dorsal fin shape variation were explored at two levels: between the two recognized subspecies and among the geographic units described for South American and Kerguelen populations. All analyses were interpreted cautiously, considering sample-size differences among groups.

## 3. Results

### 3.1. Dorsal Fin Shape

**Figure 2:**
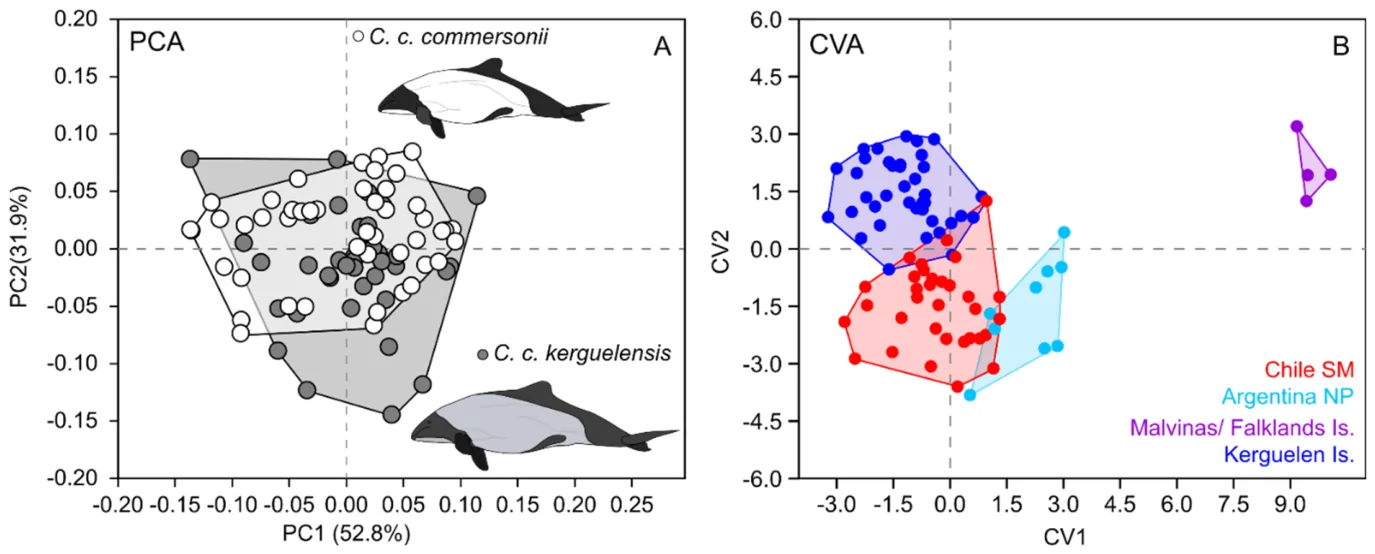
(**A**) Principal components analysis of dorsal fin shape by subspecies, *C. c. commersonii* shown with white circles with a translucent white polygon; *C. c. kerguelenensis* as dark-gray circles with a dark-gray polygon; (**B**) Canonical Variate Analysis of dorsal fin shape among groups, Chile SM = red, Argentina NP = light blue, Falkland/Malvinas Islands = purple, Kerguelen Islands = blue. Points are individuals and polygons delineate each group. Dashed lines indicate zero values on the respective axes.

Principal Components Analysis (PCA) revealed a partial overlap in morphospace and differences in morphological variability between the Kerguelen and South American subspecies (Figure 2A), with a higher degree of shape variation observed in the Kerguelen group. The first principal component (PC1) accounted for 52.8% of the variance and mainly reflected variation in fin height and the relative width of the base, separating the more robust fins of *C. c. kerguelenensis* from those of *C. c. commersonii*. The second component (PC2) explained 31.9% of the variance and was associated with differences in the posterior margin and free span curvature. Together, PC1 and PC2 summarized 84.7% of total shape variation, indicating differences in fin morphology between subspecies. Nevertheless, Canonical Variate Analysis (CVA) indicated morphological differentiation among the four geographic groups, although some overlap was evident in canonical space (Figure 2B). The Falkland/Malvinas Islands showed the greatest separation from the remaining groups, whereas Chile (Strait of Magellan) and northern Argentina exhibited partial overlap. These visual patterns were consistent with the significant pairwise Mahalanobis distances (Table 2), but should be interpreted with caution given the relatively small sample sizes of some geographic groups.

**Figure 3:**
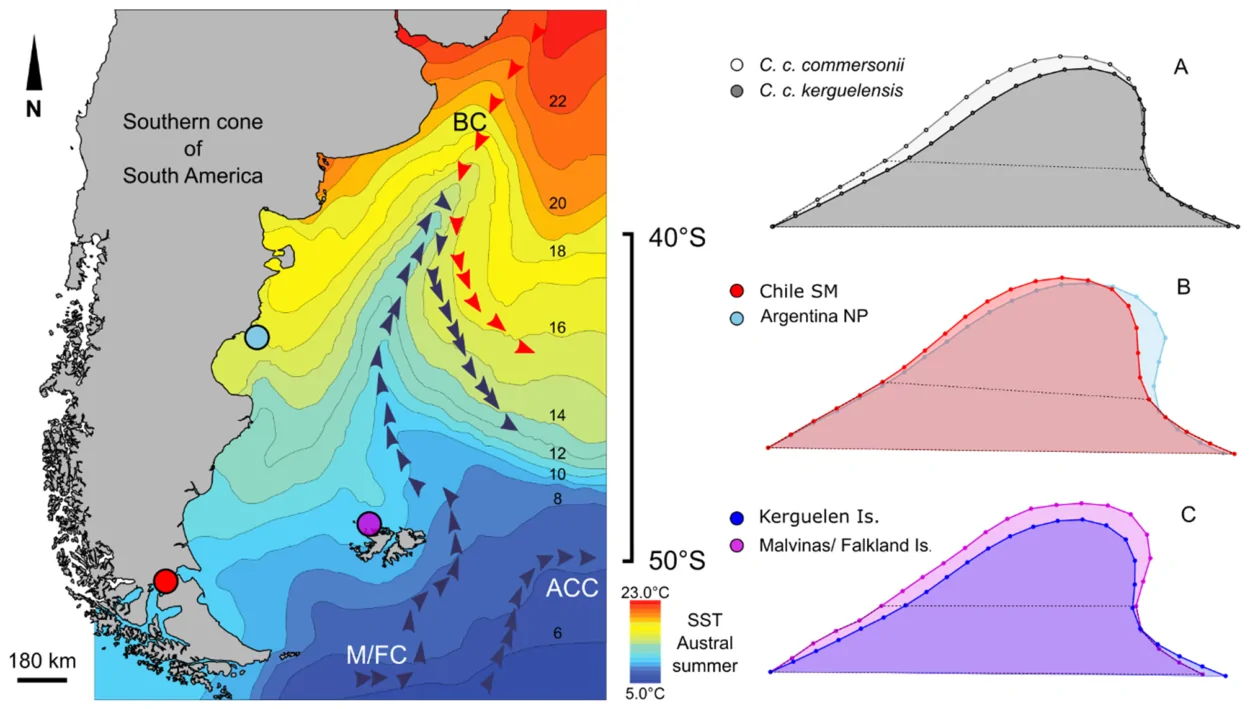
**Left panel**: Distribution map of the South American populations showing austral summer sea surface temperature (SST) and the major oceanographic currents (ACC, Antarctic Circumpolar Current; M/FC, Malvinas/Falkland Current; BC, Brazil Current). **Right panel**: Comparisons of mean dorsal fin shapes for the different geographic contrasts: (**A**) between subspecies (*C. c. commersonii* and *C. c. kerguelenensis*); (**B**) Chile (Strait of Magellan) versus Argentina (North population, Puerto Deseado); and (**C**) Kerguelen Islands versus Malvinas/Falklands Islands.Dashed lines in the right panels represent the overall mean dorsal fin shape used as a reference for visualizing shape differences.

Differences in dorsal fin shape were observed between subspecies. The Kerguelen subspecies showed a smaller and the base broader compared to the South American subspecies (Figure 3A). Variation in mean fin shape was also observed among populations. Comparing Chile (Strait of Magellan) with Argentina (North population), Magellanic dolphins tended to show a smaller, more rounded free span with a broader posterior base, whereas the northern Argentine population was characterized by a wider free span with a more pronounced posterior extension (Figure 3B). A similar pattern was observed between the Kerguelen and Malvinas/Falklands Islands: Fins from Kerguelen had a smaller free span and a wider base, while Falkland/Malvinas fins showed a wider free span extending posteriorly (Figure 3C).

### 3.2. Subspecies and Group Morphological Comparisons

Dorsal fin shape showed differences between *C. c. commersonii* and *C. c. kerguelenensis*, as indicated by Mahalanobis distance (MD = 3.3089; *p* < 0.0001; 9999 permutations). At the population level, the Malvinas/Falklands Islands showed the largest Mahalanobis distances in pairwise comparisons, with values exceeding 9 in all contrasts (Table 2). The closest similarity was observed between the Kerguelen Islands and Chile (Strait of Magellan) (MD = 3.2402; *p* < 0.001; 9999 permutations). Overall, Mahalanobis distances indicated varying degrees of separation among populations and Canonical Variate Analysis (CVA) showed separation among geographic units.

## 4. Discussion

This study documents variation in dorsal fin shape in *Cephalorhynchus commersonii* across its subantarctic range. Patterns of morphological variation were observed between subspecies and among populations, with differences in fin configuration and variability across geographic regions. These results highlight the potential of dorsal fin morphology as a trait for examining phenotypic variation in small coastal cetaceans. Commerson’s dolphin is a small coastal species characterized by high philopatry, largely driven by females [32,33]. Phylogeographic patterns reveal hierarchical levels of structure across three main regions: South America (SA, Chile-Argentina), the Falkland/Malvinas Islands (SA), and the Kerguelen Islands. The strongest differentiation occurs between Kerguelen and South America distribution range, supporting the recognition of *C. c. kerguelenensis* as distinct from *C. c. commersonii*. Genetic differences have also been reported between Chile-Argentina and the Malvinas/Falklands Islands, where gene flow is limited [27]. Within Argentina, distinct population units have also been detected based on mtDNA markers. At finer spatial scales, additional differentiation is evident within southern Patagonia, Tierra del Fuego, and the Strait of Magellan [32,34]. Overall, In South America, population genetic structure has been described at multiple spatial scales, reflecting varying degrees of connectivity among regions [27,32]. However, patterns of dorsal fin shape did not fully align with this genetic structure, suggesting that morphological variation across populations may be influenced by additional factors beyond genetic differentiation. Fin-shape patterns did not mirror population genetic structure. The greatest morphological similarity occurred between the Strait of Magellan and the Kerguelen Islands, which belong to different subspecies and are separated by approximately 8500 km with no evidence of recent genetic connectivity [27]. This may reflect similarities in environmental conditions across these regions. Morteo, Rocha-Olivares, Morteo and Weller [11] suggested that phenotypic patterns in dolphin dorsal fin shape can be influenced by multiple factors, including environmental variables linked to ecotypes and habitat as well as individual dispersal. Consistent with this view, our analysis detected similar fin shapes in *C. commersonii* from the Kerguelen Islands and the Strait of Magellan, which may be associated with lower sea surface temperatures in both regions compared with other South American and Malvinas/Falklands localities [16]. Traditional morphometrics based on linear measurements and angles has been useful for approximating dorsal fin shape and detecting individual and population differences. In *Tursiops truncatus*, for example, coastal ecotypes tend to have smaller fins, measured as a shorter distance from the anterior insertion to the posterior margin, whereas oceanic individuals show a sharper fin with a more curved posterior margin [35]. Such morphometric contrasts often accompany spatial segregation and habitat differences, producing ecotypes that differ in diet, parasite load, and group size. In our case, populations associated with islands or more open waters, such as the Malvinas/Falklands and Kerguelen Islands, displayed contrasting fin configurations even where bathymetry or habitat categories appear superficially similar. Phenotypic differences in *C. commersonii* were observed across regions. Variation in dorsal fin shape was lower in the Kerguelen Islands compared with South American populations, which may be associated with the relatively stable oceanographic conditions of this region, influenced by the Subantarctic Front and the Antarctic Circumpolar Current [36], in contrast with the more variable conditions along South America driven by interacting current systems [37]. Dorsal fins are functionally relevant structures that experience both hydrodynamic forces and physiological constraints. Previous studies have highlighted the importance of vascularization and exposed surface area in dorsal fin heat exchange [38].

The northern distributional limit of *C. commersonii* lies near Península Valdés at 41°26′ S, and the species has been recorded as far south as 40 nautical miles southeast of Cape Horn at 56°33′ S. Reports from the Drake Passage at 58° S and near Livingston Island at 61° S exist but are considered doubtful [14]. Along South America, the species appears to remain near the continental shelf and does not use deep oceanic waters. This distribution, including the presence in Kerguelen, may reflect physiological constraints. Other odontocetes occurring south of the Antarctic Convergence tend to be larger; for example, the hourglass dolphin *Lagenorhynchus cruciger* at approximately 200 cm total length and 120 kg body mass [39,40]; the spectacled porpoise *Phocoena dioptrica* at about 230 cm; and *Orcinus orca*, whose three Antarctic ecotypes exceed 530 cm and 3600 kg [41].

The Kerguelen subspecies, *C. c. kerguelenensis*, differs in coloration and body size (Goodall et al., 1988; Robineau et al. 2007) [23,42], and our results additionally reveal differences in dorsal fin configuration relative to South American populations. Similarities observed between distant regions such as the Strait of Magellan and Kerguelen may reflect the influence of shared environmental conditions, although this interpretation should be considered tentative given the limited sample size of some populations and warrants further investigation. Overall, our results show that dorsal fin shape varies across the distribution of *Cephalorhynchus commersonii*, with differences observed among both subspecies and populations. These patterns do not fully align with population genetic structure, suggesting that multiple factors may contribute to morphological variation in this species. A limitation of this study is that sex and age information were unavailable for most individuals because the analyses were based on photographs of free-ranging dolphins obtained from photo-identification catalogues and validated photographic records, precluding the evaluation of these variables. Future studies integrating genetic sex identification and longitudinal photo-identification data will help assess their potential influence on dorsal fin morphology. The observed similarities between geographically distant populations highlight the potential role of shared environmental conditions, although further studies integrating ecological, physiological, and genetic data will be necessary to better understand the drivers of this variation.

## 5. Conclusions

This study provides evidence that dorsal fin shape varies across the geographic range of Commerson’s dolphin, revealing morphological differences among both subspecies and populations. These patterns only partially correspond to previously described genetic structure, they suggest that environmental conditions may contribute to shaping phenotypic variation in this species. Our findings highlight the utility of geometric morphometrics for investigating spatial morphological variation in cetaceans and provide a framework for future studies integrating ecological, physiological, and genetic data to better understand the processes underlying population differentiation.

## Figures and Tables

**Table 2:** Pairwise group comparisons based on Mahalanobis distance (MD). * *p* < 0.001; 9999 permutation.

Locality	Argentina NP	Chile SM	Kerguelen Is.
Chile SM	4.4443 *		
Kerguelen Is.	5.2204 *	3.2402 *	
Malvinas/Falklands Is.	9.3327 *	10.5228 *	10.8726 *

## Data Availability

Data will be available by author request.
